# Facultative methanotrophs are abundant at terrestrial natural gas seeps

**DOI:** 10.1186/s40168-018-0500-x

**Published:** 2018-06-28

**Authors:** Muhammad Farhan Ul Haque, Andrew T. Crombie, Scott A. Ensminger, Calin Baciu, J. Colin Murrell

**Affiliations:** 1 School of Environmental Sciences, University of East Anglia, Norwich Research Park, Norwich, NR4 7TJ UK; 2 School of Biological Sciences, University of East Anglia, Norwich Research Park, Norwich, NR4 7TJ UK; 3 Western New York Waterfall Survey, North Tonawanda, New York USA; 4 Faculty of Environmental Science and Engineering, Babeş-Bolyai University, Cluj-Napoca, Romania

**Keywords:** *Methylocella*, Facultative methanotrophs, Natural gas, Geological methane, Biological methane, Methane monooxygenase, *mmoX*

## Abstract

**Background:**

Natural gas contains methane and the gaseous alkanes ethane, propane and butane, which collectively influence atmospheric chemistry and cause global warming. Methane-oxidising bacteria, methanotrophs, are crucial in mitigating emissions of methane as they oxidise most of the methane produced in soils and the subsurface before it reaches the atmosphere. Methanotrophs are usually obligate, i.e. grow only on methane and not on longer chain alkanes. Bacteria that grow on the other gaseous alkanes in natural gas such as propane have also been characterised, but they do not grow on methane. Recently, it was shown that the facultative methanotroph *Methylocella silvestris* grew on ethane and propane, other components of natural gas, in addition to methane. Therefore, we hypothesised that *Methylocella* may be prevalent at natural gas seeps and might play a major role in consuming all components of this potent greenhouse gas mixture before it is released to the atmosphere.

**Results:**

Environments known to be exposed to biogenic methane emissions or thermogenic natural gas seeps were surveyed for methanotrophs. 16S rRNA gene amplicon sequencing revealed that *Methylocella* were the most abundant methanotrophs in natural gas seep environments. New *Methylocella*-specific molecular tools targeting *mmoX* (encoding the soluble methane monooxygenase) by PCR and Illumina amplicon sequencing were designed and used to investigate various sites. Functional gene-based assays confirmed that *Methylocella* were present in all of the natural gas seep sites tested here. This might be due to its ability to use methane and other short chain alkane components of natural gas. We also observed the abundance of *Methylocella* in other environments exposed to biogenic methane, suggesting that *Methylocella* has been overlooked in the past as previous ecological studies of methanotrophs often used *pmoA* (encoding the alpha subunit of particulate methane monooxygenase) as a marker gene.

**Conclusion:**

New biomolecular tools designed in this study have expanded our ability to detect, and our knowledge of the environmental distribution of *Methylocella*, a unique facultative methanotroph. This study has revealed that *Methylocella* are particularly abundant at natural gas seeps and may play a significant role in biogeochemical cycling of gaseous hydrocarbons.

**Electronic supplementary material:**

The online version of this article (10.1186/s40168-018-0500-x) contains supplementary material, which is available to authorized users.

## Background

Methane is an integral component of the global carbon (C) cycle and one of the most significant contributors to climate change since it has a global warming potential approximately 34 times greater than carbon dioxide [1]. Atmospheric concentrations of methane have been steadily rising since the Industrial Revolution, currently around 1.8 ppm by volume [2]. Approximately 70% of the total 500 to 600 million tonnes methane emitted [2] is new methane, i.e. produced by methanogens during microbial degradation of organic matter, largely under anaerobic conditions. This biological process is particularly prevalent in wetlands, landfills, rice paddies, the rumen of cattle and the hindgut of termites. The remaining 30% of the methane released into the atmosphere arises from the thermogenic decomposition of fossil organic material to geological methane and other gases collectively known as natural gas [2]. Natural gas consists usually of geological methane and substantial amounts of the short chain alkanes ethane, propane and butane [3], and from subsurface reservoirs it reaches the surface of the Earth through natural seepage or mining and extraction activities.

Globally, geological methane from natural gas seeps is the second largest natural source, after wetlands, and apart from methane, it also contributes up to 3–6 million tonnes of climate-active ethane and propane per year [4]. Seepage of natural gas occurs in a wide range of environments, e.g. hydrocarbon-prone sedimentary basins, both as visible features including dry gas seeps and mud volcanoes, or in the marine realm as hydrothermal vents or shallow marine methane seeps, but also as invisible microseepage [3, 5–8]. Volcanic and geothermal systems, hot and cold springs or alkaline soda lakes, may also release non-negligible amounts of methane [9–11]. Spectacular releases of natural gas are observed at the Eternal Flame Falls in Chestnut Ridge Park, New York, where seep gas contains methane plus 35% ethane and propane [12]. Gas releases caused by human activity range from incidents such as the Deepwater Horizon disaster of 2010 (where 170,000 tonnes of natural gas escaped to the marine environment) to operational releases including leaking gas pipelines and coal mining activities [13]. Unintentional releases of natural gas are widespread and likely to increase, especially with the exploitation of unconventional resources including shale gas extraction, with associated concerns of environmental pollution and climate change [14–17].

Although a vast amount of methane escapes to the atmosphere, much more would escape if it were not for the activity of microbes that consume methane. Over half of the methane produced by methanogens in wetlands has been reported to be consumed by aerobic methanotrophs [18–20]. These methane-oxidising bacteria are a remarkable group of microbes that use methane as their sole source of carbon and energy. Aerobic methanotrophs are mainly Gram-negative bacteria of the classes *Alphaproteobacteria* and *Gammaproteobacteria*. They are usually obligate methanotrophs, unable to grow on other alkanes and multi-carbon compounds, except for only a few strains, which can grow on acetate and ethanol [21, 22]. During the metabolism of methane by aerobic bacteria, the first step is the oxidation of methane to methanol, which is catalysed by one of two enzymes: a membrane-bound, copper-containing particulate methane monooxygenase (pMMO) or a diiron centre containing soluble methane monooxygenase (sMMO) [23–26]. Both conventional enrichment experiments and cultivation-independent studies indicate that obligate methanotrophs are widespread in the environment, especially in areas rich in methane [27–30].

Specialised microbes growing on other gaseous alkanes such as ethane or propane (propanotrophs) have also been characterised, including metabolically versatile *Actinobacteria* (*Rhodococcus* and *Mycobacterium*) [31–33], *Gammaproteobacteria* (*Psuedomonas*) [34] or *Betaproteobacteria* (*Thauera*) [35, 36] that grow on many multi-carbon compounds. Most propanotrophs contain a propane monooxygenase enzyme (PrMO) with similarities to sMMO but do not grow on methane [37, 38]. An exciting development in the study of biological methane oxidation was the isolation of facultative methanotrophic strains of the genus *Methylocella* from acidic peat, tundra and forest soils [39–41]. These unusual methanotrophs grow on methane as well as some multi-carbon compounds including acetate, pyruvate, succinate and gluconate [42, 43]. *Methylocella* belong to alphaproteobacterial family *Beijerinckiaceae* containing generalist organotrophs (e.g. *Beijerinckia indica*), facultative methanotrophs (e.g. *Methylocella silvestris*) and obligate methanotrophs (e.g. *Methylocapsa acidiphilia*) [44]. Examination of the *Methylocella silvestris* BL2 genome revealed that unlike most methanotrophs, *Methylocella* did not contain genes for pMMO but oxidised methane using the sMMO enzyme only [45]. Surprisingly, genes encoding a PrMO were also identified in the *Methylocella silvestris* BL2 genome. Crombie and Murrell [46] reported for the first time that *Methylocella silvestris* BL2 derives growth benefits from oxidising methane and propane simultaneously using two distinct enzymes, sMMO and PrMO. This discovery overturned the dogma that degradation of methane and other alkane components of natural gas requires different groups of microbes.

The unique metabolic capabilities of *Methylocella* have profound implications for the biological consumption of natural gas in the environment. *Methylocella*, being able to use most components of natural gas for growth, may have a competitive edge over less versatile obligate methanotrophs and propanotrophs in environments rich in natural gas. As little is known about the distribution of *Methylocella* in the environment, the purposes of this study were to improve molecular methods for detection of *Methylocella* in environmental samples and to test the hypothesis that *Methylocella*-like facultative methanotrophs are prevalent in thermogenic, natural gas seep environments.

## Results and discussion

### Methanotrophs present at biogenic methane and natural gas seep environments

Since *Methylocella* species are the only methanotrophs known to use methane and the other components of natural gas such as ethane and propane simultaneously [46], we hypothesised that *Methylocella* may be abundant in environments exposed to thermogenic natural gas seeps. For centuries, natural gas seeps have been reported in New York state, part of the Appalachian Basin in the USA [47–49], exemplified by towns such as Gasport (Niagara County), named in 1826. Many of these seeps emit natural gas, which can be ignited (Additional file 1: Figure S1). The best-known example of such seep sites is the “Eternal Flame” in Chestnut Ridge National Park [12]. We explored many such documented and undocumented thermogenic gas seeps in this region for sample collection and found that methane and also considerable amounts of ethane and propane were present in gas collected directly from the seep sites (Additional file 1: Table S1).

**Fig. 1:**
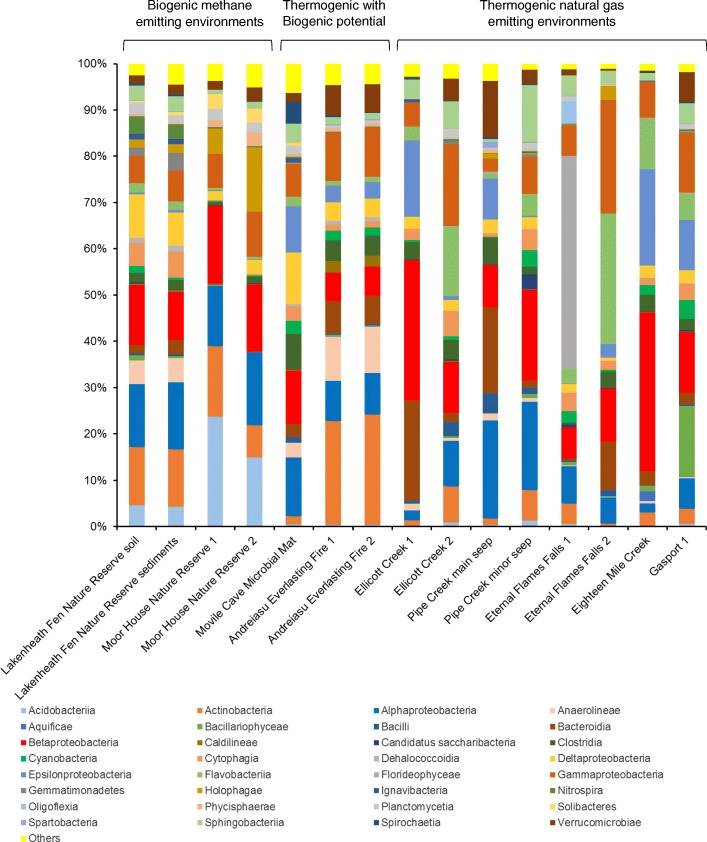
Relative abundance (%) of dominant bacterial classes in different environments as revealed by 16S rRNA gene sequencing. Amplicon sequencing was performed on DNA samples from environments exposed to biogenic methane and/or natural gas emissions

Libraries of 16S rRNA genes were generated from DNA extracted from samples from diverse environments known to be exposed to biogenic methane or thermogenic natural gas seeps (Fig. 1). Illumina Mi-Seq yielded 617,613 good quality sequence reads in total from 15 samples, averaging 41,178 sequences per sample (Additional file 2: Table S2). Sequence analysis showed that out of 20 phyla at an abundance of higher than 1% in one or more of the samples, *Proteobacteria* (alpha, beta and gamma), *Actinobacteria*, *Acidobacteria*, *Bacteroidetes*, *Chloroflexi*, *Firmicutes*, *Planctomycetes* and *Verrucomicrobia* contributed substantially to the bacterial communities (Fig. 1). Dominant families included *Acidobacteriaceae* (Moor House Nature Reserve), *Comamonadaceae* (Ellicott Creek and Eternal Flame Falls), *Flavobacteriaceae* (Ellicott Creek and Eternal Flame Falls, Eighteen Mile Creek and Gasport), *Hyphomicrobiaceae* (Lakenheath Fen Nature Reserve), *Porphyromonadaceae* (Ellicott Creek, Pipe Creek), *Mycobacteraceae* (Andreiasu Everlasting Fire), *Verrucomicrobiaceae* (Pipe Creek, Gasport), *Campylobacteraceae* (Movile Cave mat) and *Beijerinckiaceae* (Pipe Creek). When analysed at the genus level, 16S rRNA gene sequences were resolved into 1062 operational taxonomic units (OTUs), of which 129 OTUs were found at an abundance of higher than 1% in at least one sample (Additional file 2: Table S2).

**Fig. 2:**
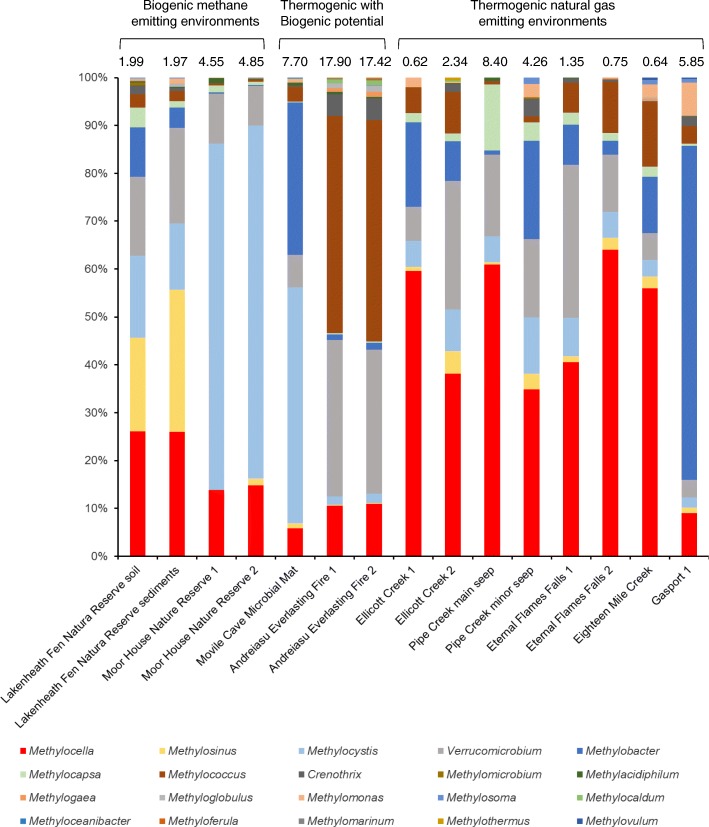
Relative abundance (%) of methanotrophic bacteria in environmental samples as revealed by 16S rRNA gene sequencing. Amplicon sequencing was performed on DNA samples from environments exposed to biogenic methane and/or natural gas emissions. The proportion (%) of the combined methanotrophic population in each environment is shown above each bar, based on the abundance of 16S rRNA gene sequences of known methanotrophs (data filtered from Additional file 2: Table S2)

Detailed analysis of 16S rRNA gene amplicons revealed that of the methanotrophs, the genera *Methylobacter*, *Methylocella*, *Methylococcus*, *Methylocystis*, *Methylosinus* and possibly *Verrucomicrobia* dominated all samples (Fig. 2). Methanotrophs accounted for 0.62–17.90% of the total bacterial population present in all 15 environmental samples (Fig. 2). *Methylocystis*, *Methylosinus*, *Methylocella* and *Verrucomicrobia* dominated in samples from sites of biogenic methane emissions (Lakenheath Fen Nature Reserve and Moor House Nature Reserve) (Fig. 2). *Methylococcus*, *Verrucomicrobia* and *Methylocella* were abundant in Andreiasu Everlasting Fire (Fig. 2), a Romanian mud volcano site reported to have largely thermogenic natural gas emissions [5, 6] but with the potential of biogenic methane emissions as revealed by the presence of methanogenic archaea in nearby mud volcanoes [50]. *Methylocystis*, *Methylobacter* and *Methylocella* were the dominant methanotrophs in microbial mat samples from Movile Cave, a very unusual, dark chemoautotrophic habitat also known to contain methane from both biogenic and thermogenic activities [51–53]. Like other environmental samples tested in this study, *Verrucomicrobia* were also found in Movile Cave samples, but we are not certain if the *Verrucomicrobia* detected by 16S rRNA in these environmental samples were methanotrophic or non-methanotrophic. Many other methanotrophic genera such as *Clonothrix*, *Methylohalobius*, *Methylomagnum*, *Methylomarinovum*, *Methyloparacoccus*, *Methyloprofundus*, *Methylosarcina* and *Methyloterricola* were not found in any of the tested samples. Interestingly, the facultative methanotroph, *Methylocella*, was the most abundant methanotroph in samples from natural gas seep environments (with the exception of Gasport samples) and accounted for 25–64% of total methanotrophs in samples from thermogenic natural gas seeps of Ellicott Creek, Pipe Creek, Eternal Flame Falls and Eighteen Mile Creek (Fig. 2). *Methylocella* appeared to be the indicator methanotrophic genus in most natural gas seep sites (Additional file 1: Figure S2). *Methylocella* abundance and the proportion of ethane and propane showed a positive correlation (Spearman’s rank correlation coefficient = 0.80, *P* value = 0.03). Metabolic versatility and the capability of utilising ethane and propane along with methane may confer an advantage over obligate methanotrophs, allowing *Methylocella* to colonise environments exposed to propane and ethane as well as methane. *Methylocella silvestris* BL2 exhibits higher growth rates and carbon conversion rates when grown under a mixture of propane and methane as compared to growth on either of these gases alone [46]. The presence of ethane and propane alongside methane at certain sites (Additional file 1: Table S1) and the abundance of *Methylocella* in those environments tested here supports our hypothesis that *Methylocella* may have a competitive advantage over obligate methanotrophs in natural gas seep sites.

### Distribution and abundance of *Methylocella* in different environments

Since 16S rRNA gene taxonomy might not distinguish between methanotrophic and non-methanotrophic members of the *Beijerinckiaceae* family (Additional file 1: Table S3) [44], we developed a *Methylocella*-specific PCR assay targeting *mmoX* (encoding the sMMO active site subunit) to study the distribution of *Methylocella* at various sites. The use of probes targeting a bacterial functional gene rather than the 16S rRNA gene enables a much more sensitive evaluation of microbial diversity in complex environments as it limits the investigation to the functional group being studied [54]. PCR conditions for *Methylocella*-specific *mmoX* were optimised and validated with DNA from pure cultures of known methanotrophs (Additional file 1: Figure S3); including a newly isolated strain *Methylocella silvestris* TVC [55]. DNA extracted from various environmental samples was PCR-screened for *Methylocella*-specific *mmoX* genes (Table 1). Of the 31 samples originating from diverse locations where there are biogenic methane and/or thermogenic natural gas emissions, *Methylocella*-specific *mmoX* gene PCR products were detected in 25 samples (Table 1), from both types of environments, i.e. biogenic methane emitting and/or thermogenic natural gas emitting. *Methylocella*-specific *mmoX* was detected in samples from biogenic methane-emitting environments with slightly acidic to moderately acidic pH (e.g. Lakenheath Fen Nature Reserve and Moor House Nature Reserve) and was detected in all the samples from natural gas-emitting sites regardless of pH (Table 1). There are a few environments, e.g. Movile Cave, which have previously been reported to be negative for *Methylocella* [56], but we now detect *Methylocella*-specific *mmoX* from wall scrapings in this environment (Table 1), in agreement with a recent metagenomics study [57]. This suggests that our newly designed PCR assay for *mmoX* showed better sensitivity and specificity for *Methylocella* as compared to the previously reported assay [56]. Another PCR assay to detect *mmoX* of *Methylocella* was described earlier, but the authors were unable to show any *Methylocella*-specific *mmoX* amplified from environmental samples [58]. Specificities of the new primers and an optimised protocol to detect *Methylocella*-specific *mmoX* genes in DNA from environmental samples reported here were verified by constructing *mmoX*-amplicon clone libraries and Illumina amplicon sequencing.

**Table 1:** Detection of *Methylocella* using a functional gene-based PCR assay

Sampling location (country)	Coordinates	Sampling sub-site (nature of sample, pH)	Methane source	*Methylocella*-specific *mmoX*
Ellicott Creek 1, Amherst (New York, USA)	42.9687 N, 78.7475 W	Around main seep (water and sediments, 6.0)	Thermogenic natural gas	Yes^2^
Ellicott Creek 2, Amherst (New York, USA)	42.9687 N, 78.7475 W	Few meters away from main seep (water and sediments, 6.0)	Thermogenic natural gas	Yes^2^
Pipe Creek main seep, West Falls (New York, USA)	42.7042 N, 78.6812 W	A large seep with vigorous gas outflow (water, sediments and soil, 6.0)	Thermogenic natural gas	Yes^2^
Pipe Creek minor seep, West Falls (New York, USA)	42.7042 N, 78.6812 W	A small seep with less vigorous gas outflow (water and sediments, 6.0)	Thermogenic natural gas	Yes^2^
Eternal Flame Falls 1, Chestnut Ridge (New York, USA)	42.7014 N, 78.7511 W	Main falls (water and sediments, 6.0)	Thermogenic natural gas	Yes^2^
Eternal Flame Falls 2, Chestnut Ridge (New York, USA)	42.7014 N, 78.7511 W	Pool below the falls (filamentous material and water, 6.0)	Thermogenic natural gas	Yes
Gasport 1, Gasport (New York, USA)	43.1977 N, 78.5726 W	Around minor seeps in Gasport stream (water and sediments, 7.0)	Thermogenic natural gas	Yes^2^
Gasport 2, Gasport (New York, USA)	43.1977 N, 78.5726 W	Bed of Gasport stream not covered with water (sediments, 7.0)	Thermogenic natural gas	Yes
Eighteen Mile Creek, N Evans (New York, USA)	42.6963 N, 78.9365 W	Edge of the stream (water and sediments, 6.0)	Thermogenic natural gas	Yes^2^
Andreiasu Everlasting Fire 1 (Romania)	45.7507 N, 26.8330 E	Around gas seep (mud, 8.2)	Thermogenic natural gas^1^	Yes^2^
Andreiasu Everlasting Fire 2 (Romania)	45.7506 N, 26.8330 E	Few meters away from gas seep (dry soil, 8.2)	Thermogenic natural gas^1^	Yes
Beciu mud volcano 1 (Romania)	45.3853 N, 26.7163 E	Edge of mud volcanoes (mud, 6.4)	Thermogenic natural gas^1^	Yes^2^
Beciu mud volcano 2 (Romania)	45.3851 N, 26.7160 E	Crater of mud volcanoes (water, 7.2)	Thermogenic natural gas^1^	Yes
Paclele Mari mud volcano 1 (Romania)	45.3396 N, 26.7073 E	Edge of mud volcanoes (mud, 8.3)	Thermogenic natural gas^1^	Yes^2^
Paclele Mari mud volcano 2 (Romania)	45.3395 N, 26.7072 E	Edge of mud volcanoes (mud, 8.3)	Thermogenic natural gas^1^	Yes
Paclele Mici mud volcano 1 (Romania)	45.3582 N, 26.7124 E	Crater of mud volcanoes (mud, 8.6)	Thermogenic natural gas^1^	Yes^2^
Paclele Mici mud volcano 2 (Romania)	45.3582 N, 26.7123 E	Crater of mud volcanoes (mud, 8.1)	Thermogenic natural gas^1^	Yes
Lakenheath Fen Nature Reserve soil (Thetford, UK)	52.4483 N, 0.5288 E	(Peat soil and water, 6.2)	Biogenic methane	Yes^2^
Lakenheath Fen Nature Reserve sediments (Thetford, UK)	52.4483 N, 0.5288 E	(Sediments and water, 6.5)	Biogenic methane	Yes^2^
Moor House Nature Reserve 1 (Pennine Hills, UK)	52.4483 N, 0.5288 E	Eroded patches called gullies (peat soil and water, 4.0)	Biogenic methane	Yes^2^
Moor House Nature Reserve 2 (Pennine Hills, UK)	52.4483 N, 0.5288 E	Non-gullies peat soil (peat soil and water, 4.0)	Biogenic methane	Yes
Movile Cave microbial mat (Mangalia, Romania)	43.8256 N, 28.5605 E	Lake (microbial mat and water, 7.3)	Both biogenic and thermogenic	Yes^2^
Movile Cave sediments (Mangalia, Romania)	43.8256 N, 28.5605 E	Lake (sediment and water, 7.6)	Both biogenic and thermogenic	Yes
Movile Cave scrapings (Mangalia, Romania)	43.8256 N, 28.5605 E	Air bell walls (soft solid material from walls, 7.3)	Both biogenic and thermogenic	Yes^2^
Church Farm soil 1 (Bawburgh, UK)	52.6167 N, 1.1667 E	(Soil, 7.0)	Biogenic methane	No
Church Farm soil 2 (Bawburgh, UK)	52.6167 N, 1.1667 E	(Soil, 7.0)	Biogenic methane	No
Strumpshaw landfill (Norfolk, UK)	52.6027 N, 1.4791 E	Soil biofilter from a closed landfill (soil, 7.0)	Biogenic methane	Yes^2^
Stiffkey Fen and Salt Marshes 1 (Norfolk, UK)	52.9650 N, 0.9253 E	(Soil, 7.0)	Biogenic methane	No
Stiffkey Fen and Salt Marshes 2 (Norfolk, UK)	52.9650 N, 0.9253 E	(Soil, 7.0)	Biogenic methane	No
Warham Salt Marsh (Norfolk, UK)	52.9617 N, 0.89667 E	Sulphur enriched salt marsh (wet soil, 7.2)	Biogenic methane	No
Warham Salt Marsh (Norfolk, UK)	52.9617 N, 0.89667 E	Iron-enriched salt marsh (wet soil, 6.8)	Biogenic methane	No

^1^These sites have been reported to have largely thermogenic natural gas emissions [5, 6] but with potential for biogenic methane [50]

^2^*Methylocella*-specific *mmoX* verified by the construction of clone libraries from PCR products and sequencing of cloned *mmoX* fragments

Previously, only a few cultivation-dependent studies [39–41, 59–61] and cultivation-independent studies (for example [56, 62–67]) have detected *Methylocella* in a relatively small number of environments. *Methylocella* had been reported in many studies to be abundant in acidic soils [68], and the known *Methylocella* species had been isolated only from acidic soil environments including peat bogs, forest and tundra soils [39–41]. Their abundance in acidic environments may be due to the ability of *Methylocella* to use readily available acetate [42], a major intermediate of carbon turnover in these soils [42, 69]. Rahman et al. [56] reported for the first time that *Methylocella* are not limited to acidic environments as they detected *Methylocella*-specific *mmoX* in the alkaline environments of Lonar Lake (pH 10). Here, we also confirmed that the distribution of *Methylocella* is not limited to acidic environments as *mmoX* of *Methylocella* was detected in all environmental samples from thermogenic natural gas-emitting sites of acidic and basic pH (Table 1), possibly because of the metabolic flexibility and ability of *Methylocella* to utilise methane and propane in the environments where these gases co-occur. Our results show that *Methylocella* thrive best in environments with thermogenic natural gas emissions under various pH conditions (Table 1, Fig. 2).

Our results show that *Methylocella* not only dominated natural gas seep sites but they are also abundant in other environments (Fig. 2) confirming previous observations [56] that *Methylocella*-like facultative methanotrophs are widespread and abundant. Frequently, molecular ecology studies of methanotrophs have targeted the *pmoA* gene [18, 27, 28] encoding a key subunit of pMMO. *Methylocella* is unusual because it lacks pMMO and instead uses sMMO to oxidise methane [45]. The abundance of *Methylocella* in the different environments tested in this study reveals that facultative methanotrophs may have been overlooked in many cultivation-independent studies that targeted only *pmoA*. The use of both *pmoA* and *mmoX*, as genetic markers for ecological studies, is therefore important to avoid underestimating the diversity and abundance of methanotrophs in the environment. More methanotrophs that only contain sMMO and lack pMMO are being discovered [70, 71]. Therefore, there is a need to re-examine functional gene primers targeting *mmoX* to detect all methanotrophs containing only sMMO.

**Fig. 3:**
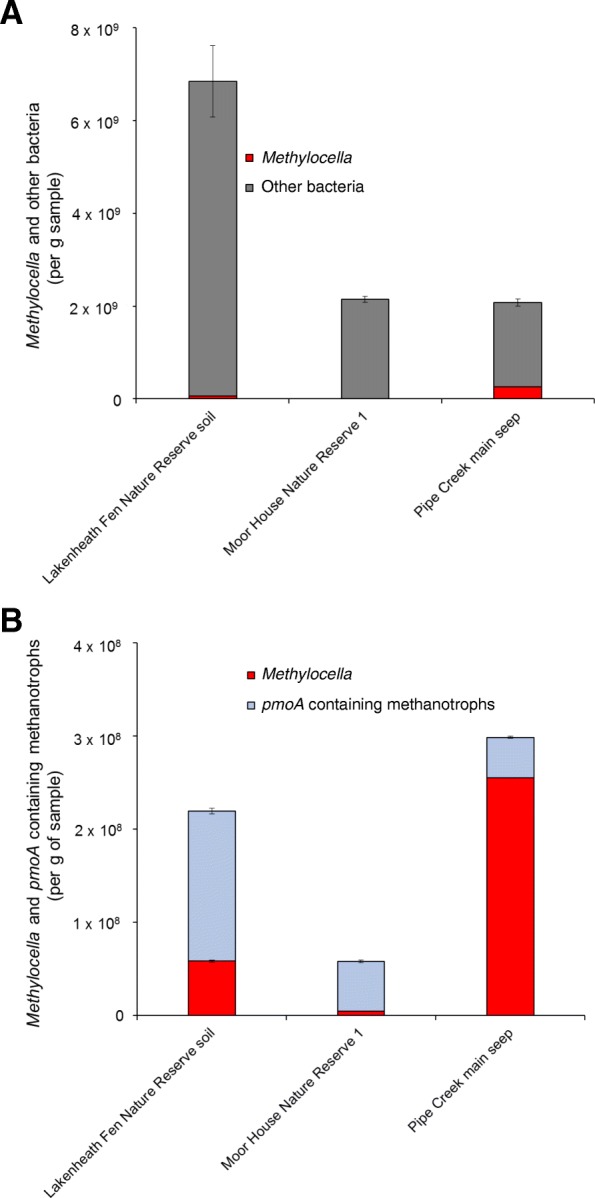
Abundance of *Methylocella* in relation to total bacteria (**a**) and *pmoA*-containing methanotrophs (**b**). Bacterial populations were enumerated by qPCR of 16S rRNA (for total bacteria), *Methylocella*-specific *mmoX* (for *Methylocella*) and *pmoA* (for *pmoA*-containing methanotrophs) genes on environmental DNA samples. *Methylocella* cell numbers equate to *Methylocella*-specific *mmoX* gene copies, whereas bacteria and *pmoA*-containing methanotrophs were assumed to contain two 16S rRNA or *pmoA* gene copies per cell. Bacteria other than *Methylocella* were enumerated by subtracting *Methylocella* from total bacterial cell numbers. Error bars represent the propagated errors based on standard deviation of triplicate samples

In addition to the 16S rRNA amplicon sequencing data, *Methylocella* abundance was also estimated by a newly developed qPCR assay targeting the *Methylocella*-specific *mmoX*. The abundance of *Methylocella* detected in selected environmental samples varied from 4.59 (± 0.19) × 10^6^ (Moor House Nature Reserve, UK) to 2.55 (± 0.06) × 10^8^ cells g^−1^ sample (Pipe Creek main seep, New York, USA) (Fig. 3). The abundance of *Methylocella*-specific *mmoX* was an order of magnitude higher in Pipe Creek main seep samples compared to other tested samples (Additional file 1: Figure S4). In contrast, the abundance of *pmoA*-containing methanotrophs in these environmental samples varied from 3.68 (± 0.12) × 10^7^ (Movile Cave Microbial Mat) to 1.61 (± 0.30) × 10^8^ *pmoA* copies g^−1^ sample (Lakenheath Fen Nature Reserve) (Fig. 3). Remarkably, in the samples from the Pipe Creek main seep, the *Methylocella* population alone constituted 5–12% of the total bacteria or 60–85% of the total methanotroph population (as estimated by 16S rRNA gene amplicon sequencing and *Methylocella*-specific *mmoX* qPCR respectively) (Figs. 2 and 3). In comparison to peat soils previously described as favourable habitats for *Methylocella* [56, 68], the *Methylocella* population was an order of magnitude higher in the natural gas seep site of Pipe Creek.

### Phylogenetic analysis of *mmoX* from *Methylocella*

**Fig. 4:**
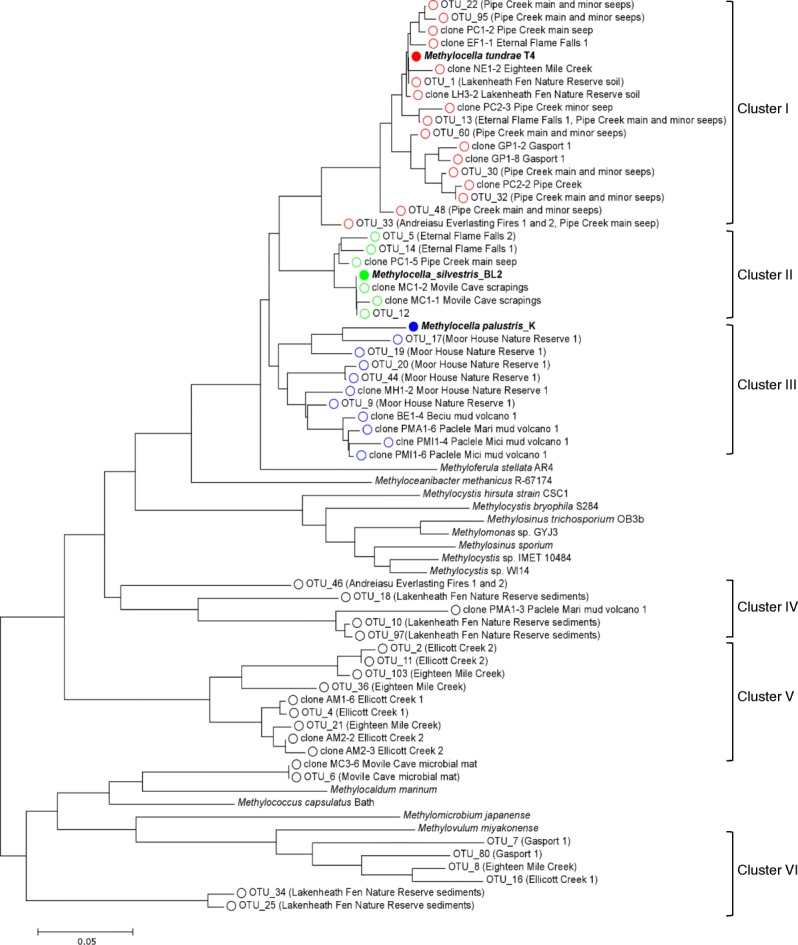
Phylogenetic tree of *Methylocella*-specific *mmoX* clones and operational taxonomical units (OTUs) retrieved by amplicon sequencing from various environmental DNA samples. *Methylocella*-specific *mmoX* clones and OTUs are grouped either around *Methylocella tundrae* T4 (red circles), *Methylocella silvestris* BL2 (green circles), *Methylocella palustris* K (blue circles) or distantly (black circles) from any known *Methylocella* strains (solid symbols). Environments where a particular OTU is abundant are shown in brackets. Partial *mmoX* sequences of representative clones and OTUs (abundance higher than 1%) and *mmoX* sequences from characterised methanotrophic bacterial strains were aligned using Mega 7.0. The optimal tree with the sum of branch length = 2.98 is shown where the evolutionary history was inferred using the neighbour-joining method taking into account a total of 323 nucleotide positions in the final dataset. The percentage (greater than 50%) of replicate trees in which the associated taxa clustered together in the bootstrap test (1000 replicates) are shown next to the branches. Scale bar represents 0.05 substitutions per site

Sequence analysis of the clone libraries generated from the *Methylocella*-specific *mmoX* PCR products obtained with different environmental DNA samples showed that most sequences are similar to known *Methylocella*-specific *mmoX* sequences in the NCBI database. These similarities ranged from 80 to 100% suggesting the possibility of novel diversity in *Methylocella*-specific *mmoX* sequences. Detailed analyses of the composition and diversity of *Methylocella* using Illumina Mi-Seq sequencing of *Methylocella*-specific *mmoX* PCR amplicons from environments exposed to thermogenic natural gas seeps and/or biogenic methane emissions were also performed. *Methylocella*-specific *mmoX* amplicon sequencing yielded 849,221 quality-filtered sequences in total for 15 samples averaging 56,615 per sample. Following sequence analysis using SwarmV2 [72], 34 OTUs with a relative abundance of higher than 1% were recovered from all samples (Additional file 3: Table S4). Phylogenetic analysis based on the DNA nucleotide sequences of the library clones and OTUs recovered from amplicon sequencing show that *mmoX* sequences clustered in several distinct clades (Fig. 4). OTUs clustering around *Methylocella tundrae* T4 and *Methylocella silvestris* BL2 were abundant in samples from Lakenheath Fen Nature Reserve soil, Pipe Creek, Eternal Flame Falls and Andreiasu Everlasting Fire while those clustering around *Methylocella palustris* K were abundant in samples from Moor House Nature Reserve samples (Fig. 4, Additional file 3: Table S4). Interestingly, a few *Methylocella*-specific *mmoX* clones and OTUs originating from Lakenheath Fen Nature Reserve, Ellicott Creek and Eighteen Mile Creek did not cluster with other known *mmoX* sequences (cluster IV and V in Fig. 4). BLAST analyses of the clones (e.g. clone AM1-6 Ellicott Creek 1, AM2-2 Ellicott Creek 2, AM2-3 Ellicott Creek 2) from this cluster further revealed their best hit to the *mmoX* from *Methylocella silvestris* BL2 but with only 81% nucleotide identity, suggesting that these environments harbour novel strains, possibly related to *Methylocella*. In some environments where *Methylocella* was not abundant, we also detected some sequences (OTUs 6, 7, 8, 25, 34 and 80) more closely related to *mmoX* from other methanotrophs. These false positives made up approximately 10% (less than 5% in clone libraries) of the total sequences reads (Fig. 4). However, this non-*Methylocella mmoX* OTU appeared to be a dominant taxon (72%) based on *mmoX* amplicon sequencing in Eighteen Mile Creek sample (Fig. 4, Additional file 3: Table S4). Therefore, a clone library or amplicon sequencing analysis should be performed to validate the results of *Methylocella*-specific *mmoX* PCR or qPCR. Phylogenetic clustering of the sequences from clone libraries and amplicon sequencing from the same samples was remarkably congruent (Fig. 4, Additional file 3: Table S4). Comparison of the different environments revealed that Pipe Creek natural gas seep was the most diverse in terms of *Methylocella*-specific *mmoX* (Additional file 3: Table S4). Although it was not possible to link *Methylocella*-specific *mmoX* diversity with either biogenic methane-emitting environments or thermogenic natural gas-emitting environments, this phylogenetic analysis suggests the possibility of novel diversity in *Methylocella*-specific *mmoX* sequences and has suggested several target sites for future isolation of new *Methylocella* strains.

## Conclusions

New biomolecular tools designed in this study have expanded our knowledge of the environmental distribution of the facultative methanotroph *Methylocella*. *Methylocella*-like facultative methanotrophs are particularly abundant at natural gas seeps and may therefore play a significant role in biogeochemical cycling of these gaseous alkanes. This study is timely since the release of natural gas into the environment globally will increase considerably with the exploitation of unconventional sources of oil and gas. A detailed mechanistic understanding of how *Methylocella*-like facultative methanotrophs mitigate these fugitive gases can now be undertaken using the tools and knowledge obtained in this study. In situ estimates of the activity of *Methylocella* oxidising methane and other alkanes simultaneously at natural gas seeps are now required to determine their impact on the cycling of these atmospheric trace gases.

## Methods

### Chemicals and reagents

All chemicals and reagents (purity > 99%) were obtained from Sigma-Aldrich unless otherwise stated. Buffers, culture media and solutions were prepared in ultra-pure water, and sterilisation was done by autoclaving (15 min, 121 °C, 1 bar) or by filtration (0.2 μm).

### Bacterial strains and growth conditions

*Methylocella* strains were grown in 20 ml diluted nitrate mineral salt (DNMS) medium, and other methanotrophs were grown using nitrate mineral salt (NMS) medium, in 120 ml serum vials, with methane (20% *v*/*v* in headspace) as the only source of C and energy, as described previously [73, 74]. The growth of liquid cultures was monitored by measuring the optical density at 540 nm.

### Sample collection and characterisation

To study the distribution of *Methylocella*-like methanotrophs, samples (soil or sediment and water) were taken from diverse environments with known emissions of biogenic methane and/or thermogenic natural gas (see Table 1 and Additional file 1: Table S1 for details). Several natural gas seeps have been reported in New York state, USA [12], and Romania [5, 6]. Five locations in New York state known to emit thermogenic natural gas were sampled in June 2017 (Table 1 and Additional file 1: Table S1). Gas bubbles from the natural gas seeps for which the concentrations of methane, ethane and propane have not been reported were also sampled for subsequent assays using gas chromatography (Table 1). Seeps from Romania mainly releasing thermogenic methane [5, 6] or potentially biogenic methane [50] were also sampled (Table 1 and Additional file 1: Table S1). The investigated features appear as mud volcanoes (Paclele Mari, Paclele Mici, Beciu) or dry seeps generating everlasting fires (Andreiasu). Samples were also taken from wetland environments known for biogenic methane emissions as a result of microbial methanogenic activity, e.g. Lakenheath Fen Nature Reserve (Norfolk, UK), Moor House Nature Reserve (Pennine Hills, UK), Church Farm soil (Bawburgh, Norfolk, UK) and Stiffkey and Warham salt marshes (Norfolk, UK). Samples from Movile Cave (Romania), an unusual habitat known to have both methanogenic and thermogenic natural gas emissions, were also obtained [53]. Two to five sub-samples were taken from each sub-site in sterile 50 ml plastic tubes, which were pooled together before DNA extraction in the lab. The pH of samples was measured in the lab using a pH meter (Jenway) using 1:5 (*w*/*w*) soil water suspensions in the case of soil or sediment samples or directly in the case of water samples.

### Measurement of gaseous hydrocarbons by gas chromatography

Alkane (C1–C3) concentrations in the gas samples at seep sites were quantified using a gas chromatograph (GC). From bubbles, 5 ml gas was taken into a syringe and injected into 30 ml pre-sealed serum vial. These vials were analysed in the lab using an Agilent 7820A GC equipped with a Porapak Q column (Supelco) coupled to a flame ionisation detector (FID) to measure methane, ethane and propane concentrations as previously described [46].

### Extraction of DNA and PCR amplification of 16S rRNA and *mmoX* genes

DNA was extracted from pure cultures of methanotrophic strains using standard methods [73]. DNA was extracted from soils, sediments or slurries using the FAST DNA spin kit for soil (MP Biomedicals), following the manufacturer’s instructions. Qubit (Invitrogen), NanoDrop (ThermoFisher Scientific) and gel electrophoresis methods were used to check quantity and quality of DNA samples.

All primers used in this study are listed in Table S5 (Additional file 1). Extracted DNA was used as the template for PCR to amplify 16S rRNA and *mmoX* genes. Initially, the absence of PCR inhibitors, such as humic acids, was confirmed by amplifying bacterial 16S rRNA genes from template DNA, extracted from all the samples, using universal primers 27F and 1492R [75]. Reactions were carried out in a 20-μl volume consisting of 10 μl PCRBIO Taq mix red (2×) (PCRBIO), 0.8 μl of each of forward and reverse primers (10 μM) and 0.8 μl of template DNAs (1 to 10 ng). The cycling conditions for PCR amplification of 16S rRNA genes were 95 °C for 3 min, followed by 30 cycles of 94 °C for 20 s, 55 °C for 20 s and 72 °C for 40 s, with a final extension at 72 °C for 5 min.

A new semi-nested PCR protocol was optimised targeting *Methylocella*-specific *mmoX* using a newly designed forward primer (mmoXLF2) and a previously designed reverse primer (mmoXLR) (Additional file 1: Table S5). For the amplification of *mmoX* genes specifically from *Methylocella* by conventional PCR, a first round of PCR was adopted using primers mmoXLF and mmoXLR, while a second round of PCR was performed with primers mmoXLF2 and mmoXLR. PCR reactions were carried out in a 20-μl volume containing 10 μl PCRBIO Taq mix red (2×) (PCRBIO), 0.8 μl of each of forward and reverse primers (10 μM) and 0.8 μl of template DNA (5 to 20 ng) or 0.8 μl first round PCR product. PCR cycling conditions for both PCR assays consisted of a touchdown programme, i.e. 95 °C for 3 min, followed by 10 cycles of 94 °C for 20 s, 70 to 61 °C (decreasing 1 each cycle) for 20 s, 72 °C for 20 s and then 25 cycles of 94 °C for 20 s, 60 °C for 20 s and 72 °C for 20 s, with a final extension at 72 °C for 5 min. For assays targeting specifically *mmoX* from *Methylocella*, PCR conditions were optimised with DNA from pure cultures of *Methylocella silvestris*, *Methylocella palustris*, *Methylocella tundrae* and from *Methylosinus trichosporium* OB3b and *Methylococcus capsulatus* Bath as negative controls (Additional file 1: Figure S3). Specificity of the primers to detect *mmoX* of *Methylocella* in environmental DNA was verified by clone library analysis using the pGEMT easy (Promega) cloning kit according to the manufacturer’s instructions (Table 1) before carrying out Illumina amplicon sequence analyses (described below). Ninety-three clones (from 17 representative samples) were sequenced and analysed. All sequences obtained were *mmoX* sequences (Table 1), of which only 5% were *mmoX* sequences related to methanotrophs other than *Methylocella*, while all other sequences obtained appeared to be *mmoX* sequences related to *Methylocella*, with 80–100% nucleotide identity to *mmoX* from *Methylocella*. Moreover, no false-positive *mmoX* sequences were detected in clone libraries from environments such as Moor House Nature Reserve and Andreiasu Everlasting Fire, where other *mmoX*-containing methanotrophs (*Methylocystis*, *Methylococcus*) were abundant (Fig. 2).

### Quantitative real-time PCR

Quantification of *Methylocella* and other methanotrophs was estimated by qPCR assays targeting *Methylocella*-specific *mmoX* (using mmoXLF2 and mmoXLR primer pair yielding an amplicon size of 389 bp) and *pmoA* (using A189F and Mb661R primer pair yielding an amplicon size of 472 bp) (see primer sequences in Additional file 1: Table S5). Quantification of 16S rRNA genes was also performed by qPCR using 519F and 907R primers (yielding an amplicon size of 388 bp). All qPCR assays were performed using StepOne Plus real-time PCR system (Applied Biosystems). Reactions were carried out in a 96-well qPCR plate (Applied Biosystems), in a total reaction volume of 20 μl, containing 10 μl of 2× SensiFAST SYBR Hi-ROX reagent (Bioline), 0.8 μl of each of forward and reverse primers (10 μM) and 0.8 μl of template DNAs or standards. Conditions for *Methylocella*-specific *mmoX* qPCR reactions consisted of an initial denaturation step at 95 °C for 3 min, followed by 40 cycles of 95 °C for 20 s, 65 °C for 30 s and 72 °C for 30 s. Specificity of amplification was determined from dissociation curves obtained by increasing 1 °C per 30 s from 65 to 90 °C and after gel electrophoresis and clone library construction from qPCR products (data not shown). Conditions for 16S rRNA gene and *pmoA* qPCR reactions consisted of an initial denaturation step at 95 °C for 3 min, followed by 40 cycles of 95 °C for 20 s, 55 °C for 30 s and 72 °C for 30 s. The gene copy numbers of *Methylocella*-specific *mmoX* and methanotrophic *pmoA* genes per microgram of template DNA were determined using calibration curves obtained from qPCR of tenfold dilution series of DNA standards (Additional file 1: Figure S5). The detection limit of the qPCR assay was ten copies of *mmoX* of *Methylocella* per 20 μl PCR reaction (Additional file 1: Figure S5). The qPCR assay was validated by spiking Warham salt marsh soil (Norfolk, UK) with known numbers of *Methylocella silvestris* BL2 and *Methylocella palustris* K cells (ranging from 10^3^ to 10^6^ cells g^−1^ soil) and by detecting the *mmoX* copies from the spiked soil (Additional file 1: Figure S6). Assuming two copies of 16S rRNA gene per cell, a single copy of *mmoX* gene per *Methylocella* cell [45] and two copies of *pmoA* per cell [30] for other methanotrophs, the abundance of methanotrophs in different samples was estimated. Controls to check for any inhibition of the qPCR assay were also performed by carrying out a qPCR assay targeting the *mmoX* of *Methylocella*, using tenfold serial dilutions of environmental DNA samples and also by doing another *Methylocella*-specific *mmoX* qPCR assay where templates were environmental DNA samples spiked with known amounts of *Methylocella silvestris* BL2 genomic DNA. Both inhibition control experiments did not show any inhibition of amplification during PCR reactions (data not shown).

### Illumina Mi-Seq sequencing of PCR amplicons

Illumina Mi-Seq sequencing of PCR amplicons obtained from environmental DNA samples and control samples with genomic DNA of *Methylocella silvestris* BL2 was performed for both 16S rRNA genes and *Methylocella*-specific *mmoX* genes. For 16S rRNA genes, universal primers 341F and 785R primers [76] targeting the V3 and V4 regions were used. PCR reactions were carried out in 25 μl containing 12.5 μl 2× PCRBIO Ultra Polymerase (PCR BIO), 1 μl of each of forward and reverse primers (10 μM) and 1 μl of template DNA. The cycling conditions were 95 °C for 3 min, followed by 25 cycles of 94 °C for 20 s, 55 °C for 20 s and 72 °C for 30 s, with a final extension at 72 °C for 5 min. Duplicate PCR reactions for each sample were pooled before purifying using a NucleoSpin Gel and PCR Clean-up Kit (Macherey-Nagel). Gel electrophoresis and a NanoDrop machine were used to assess the quantity and quality of the purified PCR products, and concentrations of all samples were adjusted to 15–20 ng per microliter. Similarly, samples were prepared for *Methylocella*-specific *mmoX* amplicon sequencing using the primers and PCR assay described above for the detection of *Methylocella* in the environment. Purified PCR products were used to prepare DNA libraries following the Illumina TruSeq DNA library protocol and sequenced (2 × 300 bp paired-end reads) at MR DNA (Shallowater, TX, USA) using the Illumina MiSeq platform.

16S rRNA sequence data were processed using MR DNA proprietary analysis pipeline (www.mrdnalab.com). Sequences were depleted of barcodes and primers then short sequences < 200 bp were removed, sequences with ambiguous base calls removed, and sequences with homopolymer runs exceeding 6 bp removed. Sequences were then denoised, and 16S rRNA gene OTUs were defined with clustering at 3% divergence (97% similarity) followed by removal of singleton sequences and chimeras [77–82]. Final OTUs were taxonomically classified using BLASTn against a curated database derived from GreenGenes [83], RDPII (http://rdp.cme.msu.edu) and NCBI (www.ncbi.nlm.nih.gov), and compiled into each taxonomic level. Abundance data of 16S rRNA gene OTUs related to *Methylocella* retrieved from the environments with known concentrations of ethane and propane (Additional file 1: Table S1) were used to calculate Spearman’s correlation coefficient at the statistical significance level of 0.05.

For *Methylocella*-specific *mmoX* sequence data, paired-end reads were merged using VSEARCH v2.6.1 [84] using default parameters. Successfully merged reads were demultiplexed, and barcodes and primers are removed using Cutadapt v1.15 [85]. Sequences were filtered for ambiguous bases and de-replicated using VSEARCH, prior to de novo clustering using SwarmV2 v2.2.2 [72, 86], with the fastidious option. Finally, chimeras were removed and sequences quality filtered using VSEARCH. Representative sequences from each OTU were extracted for subsequent phylogenetic analysis. Phylogenetic trees were constructed using Mega7.0 [87].

## Additional files

Additional file 1: Supplementary information. (PDF 1546 kb)

Additional file 2: **Table S2.** Bacterial abundance assessed by 16S rRNA. (XLSX 147 kb)

Additional file 3: **Table S4.** *Methylocella*-specific *mmoX* abundance. (XLSX 1606 kb)
