# Supplementary material for: Facultative methanotrophs are abundant at terrestrial natural gas seeps
Source: Microbiome. 2018 Jun 28;6:118. doi: 10.1186/s40168-018-0500-x (PMC6022506; doi:10.1186/s40168-018-0500-x)
Supplement: Supplementary file 1 — Supplementary information. (PDF 1546 kb) [file 40168_2018_500_MOESM1_ESM.pdf]

(Additional file 1)

Facultative methanotrophs are abundant at terrestrial natural gas seeps

Muhammad Farhan Ul Haque[1], Andrew T. Crombie[2], Scott A. Ensminger[3], Calin Baciu[4], J Colin Murrell[1]*

Authors' affiliations

[1] School of Environmental Sciences, University of East Anglia, Norwich, UK

[2] School of Biological Sciences, University of East Anglia, Norwich, UK

[3] Western New York Waterfall Survey, North Tonawanda, New York, USA

[4] Faculty of Environmental Science and Engineering, Babeş-Bolyai University, Cluj-Napoca, Romania

* Corresponding author:

J Colin Murrell, [1]School of Environmental Sciences, University of East Anglia

Norwich Research Park, NR4 7TJ, UK

E-mail: j.c.murrell@uea.ac.uk

Tel: 01603 592959 (Office); 01603 592239 (Lab)

Fax: 01603 591327

Table S1. Proportion of methane, ethane and propane from gas collected at seep sites. Percentages are shown as a proportion of the total of C1 – C3 alkanes.

Sampling site	CH_4 (%)	C_2H_6 (%)	C_3H_8 (%)	References
Ellicott Creek, Amherst, (New York, USA)	91.90 ± 0.02	6.70 ± 0.01	1.30 ± 0.02	This study
Pipe Creek, West Falls (New York, USA)	77.80 ± 0.01	15.30 ± 0.01	6.90 ± 0.01	This study
Eternal Flame Falls, Chestnut Ridge, (New York, USA)	65	$C_2H_6 + C_3H_8 = 35$		[1]
Eighteen Mile Creek, North Evans, (New York, USA)	No data available			
Gasport, (New York, USA)	92.90 ± 0.01	6.90 ± 0.01	0.20 ± 0.01	This study
Andreiasu Everlasting Fires, (Romania)	95.05	1.94	0.57	[2]
Paclele Mari mud volcano, (Romania)	94.57	1.32	0.61	[2, 3]
Paclele Mici mud volcano, (Romania)	94.53	0.05	0.01	[2, 3]
Beciu mud volcano, (Romania)	93.15	2.05	0.76	[2, 3]

Table S3. Sequence identities between 16S rRNA genes of *Methylocella* and *Beijerinckia*, two members of *Beijerinckiaceae* family.

Strain	*M. palustris*	*M. silvestris*	*M. tundrae*
B. indica DSMZ 1719	97%	97%	97%
B. indica ATCC 9039	96%	97%	97%
B. derxii DSMZ 2329	96%	97%	97%
B. mobilis DSMZ 2326	96%	96%	97%
B. doebereinerae LMG 2819	97%	96%	97%

Table S5. Primers used in this study.

Primer	Sequence (5' – 3')*	Target gene (purpose; amplicon size)	Reference
**mmoXLF2**	TGCGCGACGCCAARAAG	*Methylocella*-specific *mmoX* (conventional PCR and qPCR; ~389 bp)	This study
**mmoXLR**	CCCAATCATCGCTGAAGGAGT		[4]
**mmoXLF**	GAAGATTGGGGCGGCATCTG	*Methylocella*-specific *mmoX* (conventional PCR; ~450 bp)	[4]
**mmoXLR**	CCCAATCATCGCTGAAGGAGT		[4]
**A189F**	GGNGACTGGGACTTCTGG	*pmoA* (qPCR; ~472 bp)	[5]
**Mb661R**	CCGGMGCAACGTCYTTACC		[6]
**27F**	AGAGTTTGATCMTGGCTCAG	16S rRNA (conventional PCR; ~1450 bp)	[7]
**1492R**	TACGGYTACCTTGTTACGACTT		[7]
**341F**	CCTACGGGNGGCWGCAG	16S rRNA (conventional PCR; ~444 bp)	[8]
**785R**	GACTACHVGGGTATCTAATCC		[8]
**519F**	CAGCMGCCGCGGTAANWC	16S rRNA (qPCR; ~388 bp)	[7]
**907R**	CCGTCAATTCMTTTRAGTT		[7]

***** Equimolar mixtures at degenerate positions: R (G,A); N (G,A,T,C); M (A,C); Y (T,C); W (A,T); H (A,C,T) and V (G,A,C)

Figure S1. Natural gas burning at Pipe Creek (New York state, USA) seep. Gas bubbles (white arrows) are also visible, representing the continuous flow from the seep.

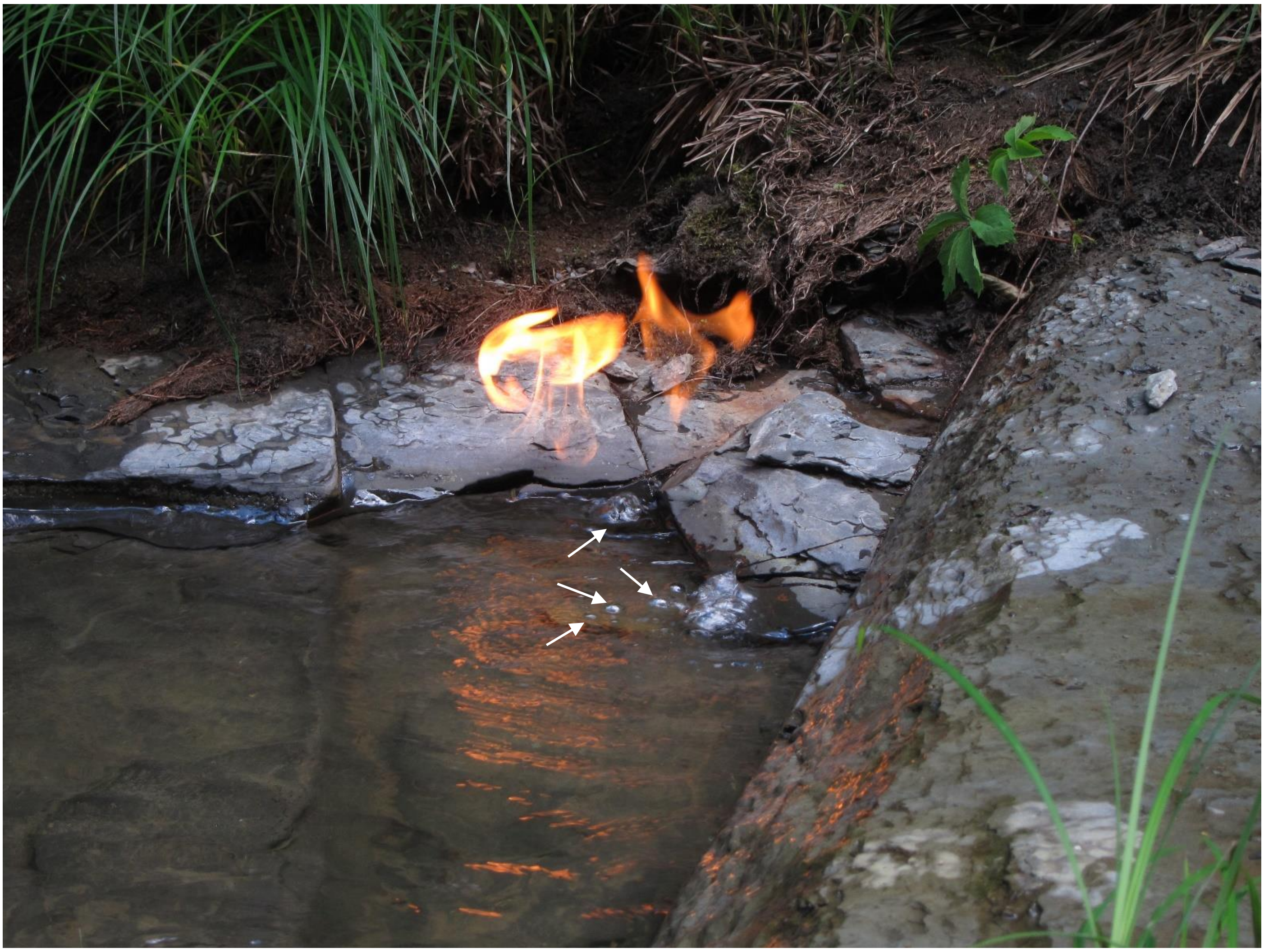

Figure S2. Principal component analysis of sample sites based on standardised abundance (%) of methanotrophic genera (data from 16S rRNA gene Illumina amplicon sequencing). Numbers 1-20 represent different genera (1 = *Methylocella*, 2 = *Methylosinus*, 3 = *Methylocystis*, 4 = *Verrucomicrobium*, 5 = *Methylobacter*, 6 = *Methylocapsa*, 7 = *Methylococcus*, 8 = *Crenothrix*, 9 = *Methylomicrobium*, 10 = *Methylacidiphilum*, 11 = *Methylogaea*, 12 = *Methyloglobulus*, 13 = *Methylomonas*, 14 = *Methylosoma*, 15 = *Methylocaldum*, 16 = *Methyloceanibacter*, 17 = *Methyloferula*, 18 = *Methylomarinum*, 19 = *Methylothermus*, 20 = *Methylovulum*). The plot was drawn using 'standardise' function (for pre-treatment of data) and then running 'PCA' analysis using PRIMER 6 software (PRIMER-E, Plymouth, UK). Two principal components (PC1 and PC2) explain over 85.6 % of total variability in data.

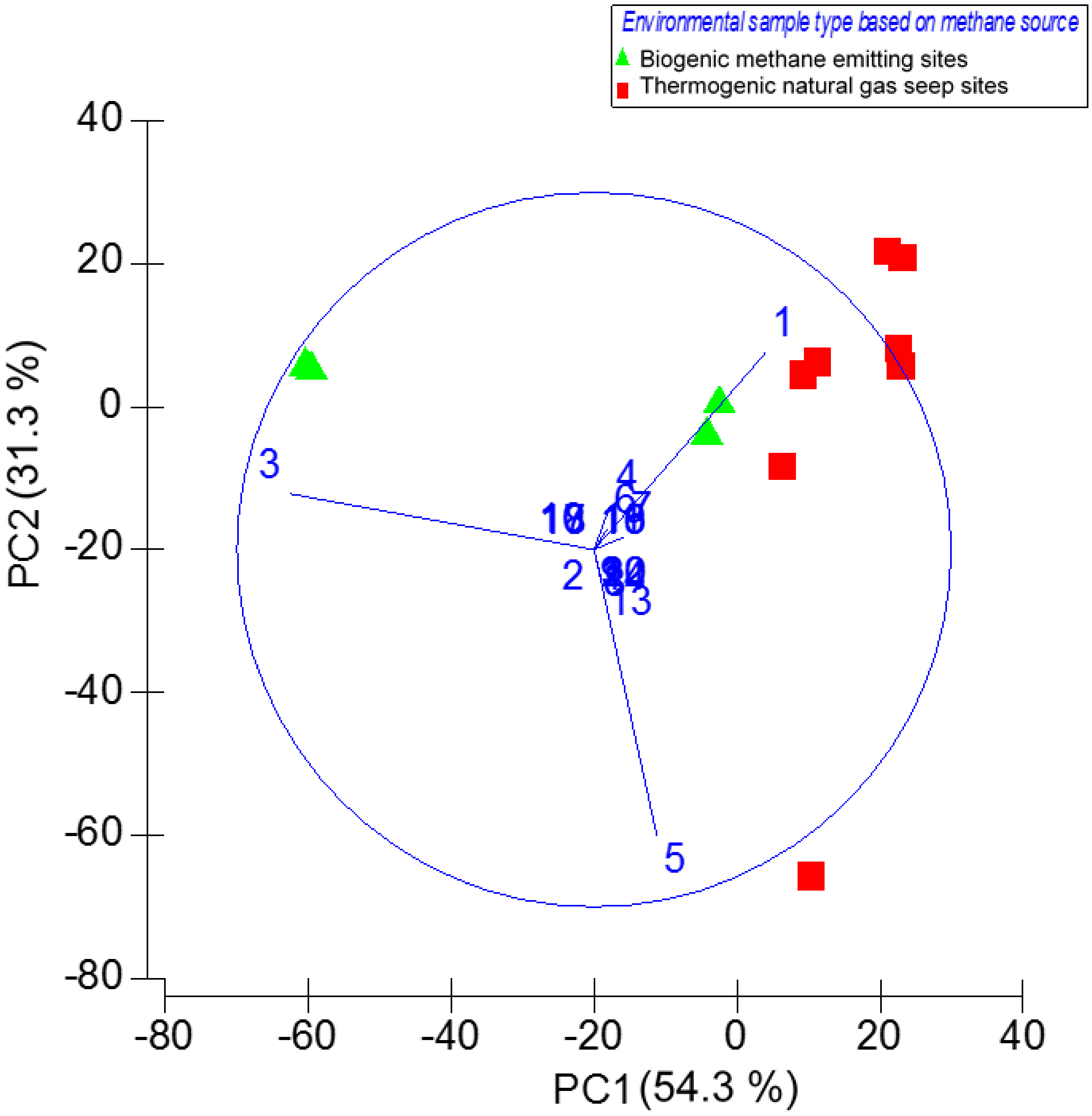

Figure S3. Validation of *Methylocella*-specific *mmoX* primers using genomic DNA from various methanotrophic bacterial strains. PCR was performed using mmoXLF2 and mmoXLR primers (yielding an amplicon of ~ 389 bp) on DNAs from *Methylocella palustris*, *Methylocella silvestris* BL2, *Methylocella silvestris* TVC, *Methylocella tundrae* T4 as positive controls (lanes 1-4 respectively) with *Methylosinus trichosporium* OB3b and *Methylococcus capsulatus* Bath (lane 6 and 7 respectively) as negative controls. Lane M represents the 1kb DNA ladder.

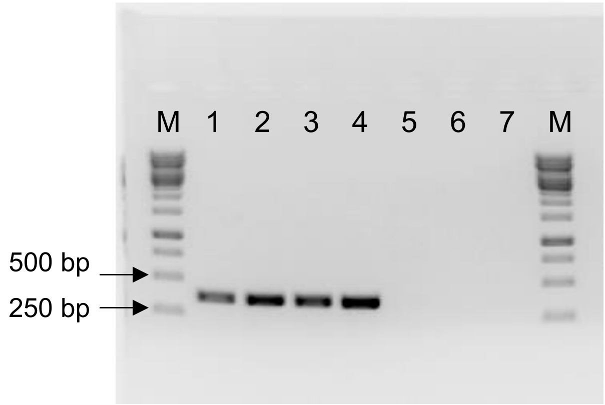

Figure S4. Quantification of 16S rRNA, *pmoA*, and *Methylocella*-specific *mmoX* genes using qPCR assays. Average copy number of the genes with errors bars showing the standard deviations of the triplicate measurements are shown.

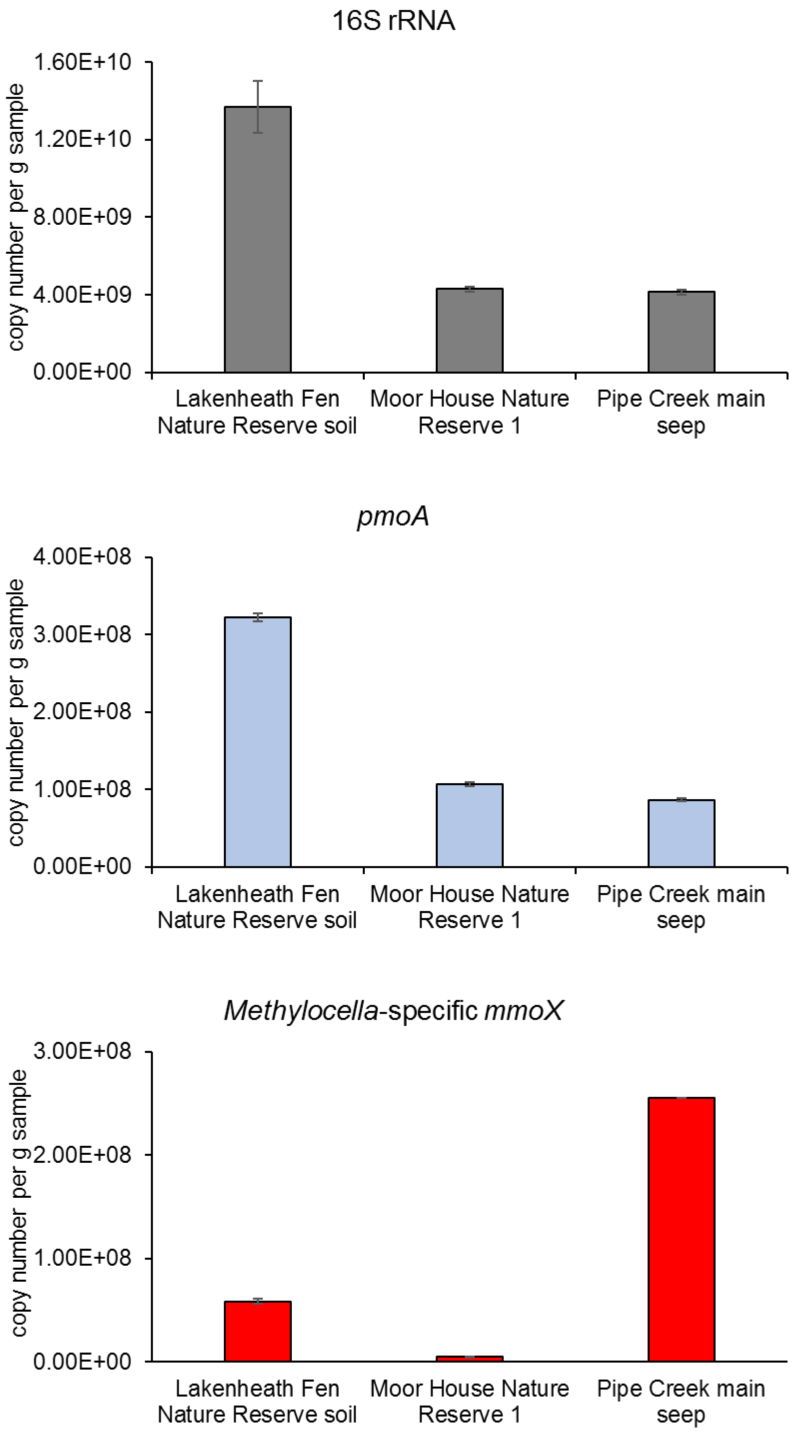

Figure S5. Calibration curves for the qPCR of various genes. Tenfold serial dilutions of standards were used as template for qPCR of 16S rRNA (A), *pmoA* (B) and *Methylocella*-specific *mmoX* (C) genes. Standards were prepare using genomic DNA from *Methylocella silvestris* BL2 (for 16S rRNA and *Methylocella*-specific *mmoX*) and *Methylococcus capsulatus* Bath (for *pmoA*). Threshold cycle (Ct) values were plotted against known copy numbers of the standards. Each point represents the average of triplicate samples with error bars (smaller than marker if not visible) showing the standard deviation.

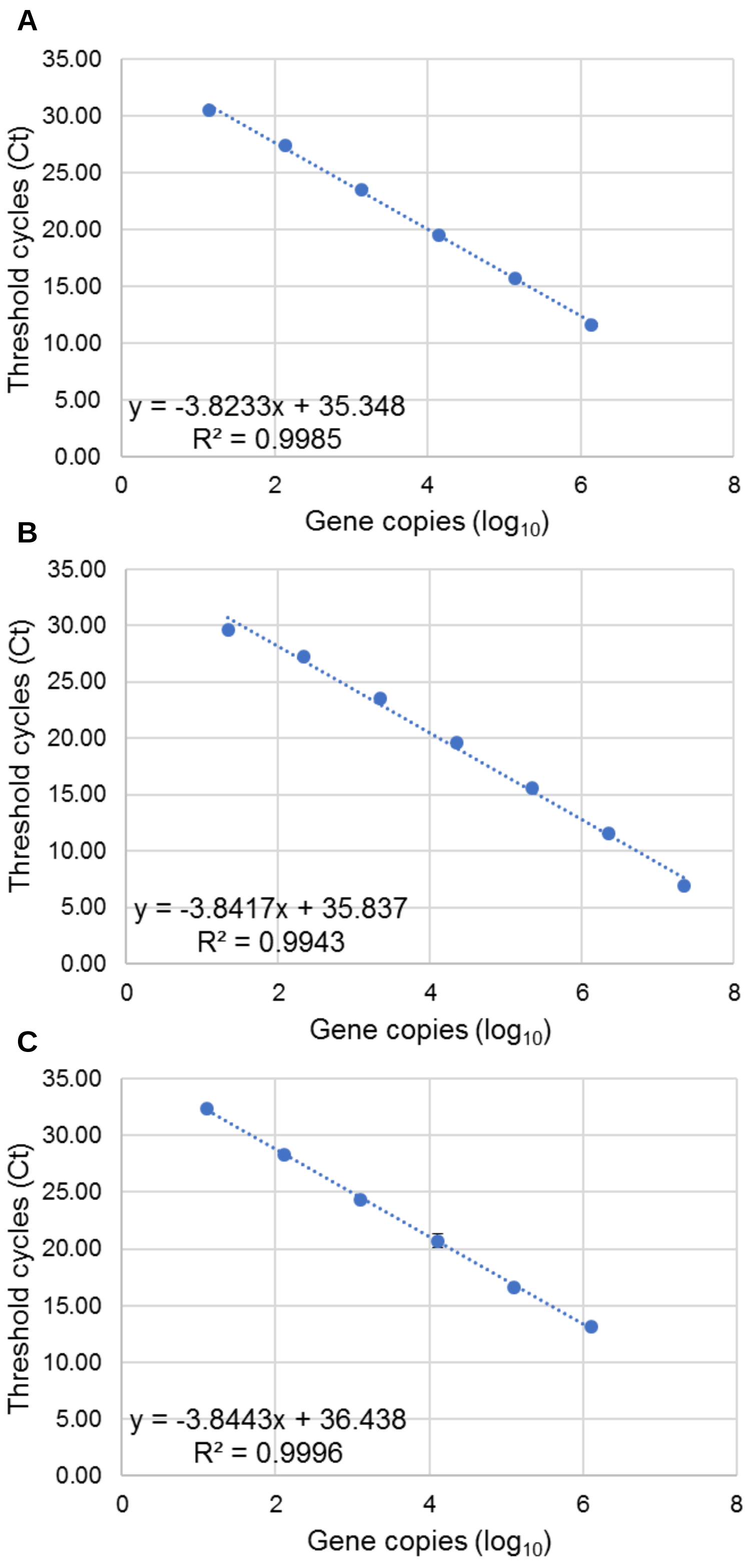

Figure S6. Detection of *Methylocella*-specific *mmoX* copies from the soil spiked with *Methylocella silvestris* BL2 (circles) or *Methylocella palustris* (squares) cells. The dotted line shows the theoretical 100% detection efficiency. Each point represents the average of triplicate samples with error bars (smaller than marker if not visible) showing the standard deviations.

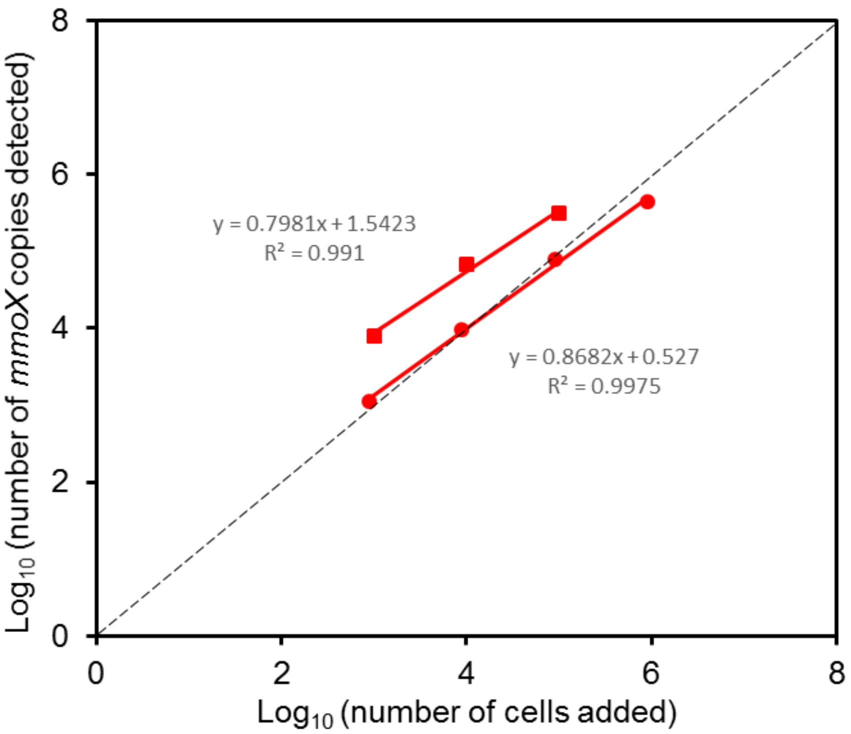

References

1. Etiope G, Drobniak A, Schimmelmann A: Natural seepage of shale gas and the origin of “eternal flames” in the Northern Appalachian Basin, USA. Mar. Pet. Geol. 2013;43:178-186.
2. Baciu C, Ionescu A, Etiope G: Hydrocarbon seeps in Romania: Gas origin and release to the atmosphere. Mar. Pet. Geol. 2018;89:130-143.
3. Etiope G, Feyzullayev A, Baciu CL: Terrestrial methane seeps and mud volcanoes: A global perspective of gas origin. Mar. Pet. Geol. 2009;26:333-344.
4. Rahman MT, Crombie A, Chen Y, Stralis-Pavese N, Bodrossy L, Meir P, McNamara NP, Murrell JC: Environmental distribution and abundance of the facultative methanotroph *Methylocella*. ISME J 2011;5:1061-1066.
5. Holmes AJ, Costello A, Lidstrom ME, Murrell JC: Evidence that particulate methane monooxygenase and ammonia monooxygenase may be evolutionarily related. FEMS Microbiol. Lett. 1995;132:203-208.
6. Costello AM, Lidstrom ME: Molecular characterization of functional and phylogenetic genes from natural populations of methanotrophs in lake sediments. Appl. Environ. Microbiol. 1999;65:5066-5074.
7. Lane DJ: 16S/23S rRNA sequencing. John Wiley & Sons, New York; 1991.
8. Klindworth A, Pruesse E, Schweer T, Peplies J, Quast C, Horn M, Glöckner FO: Evaluation of general 16S ribosomal RNA gene PCR primers for classical and next-generation sequencing-based diversity studies. Nucleic Acids Res. 2013;41:e1.
